# LSTM Networks Using Smartphone Data for Sensor-Based Human Activity Recognition in Smart Homes

**DOI:** 10.3390/s21051636

**Published:** 2021-02-26

**Authors:** Sakorn Mekruksavanich, Anuchit Jitpattanakul

**Affiliations:** 1 Department of Computer Engineering, School of Information and Communication Technology, University of Phayao, Phayao 56000, Thailand; sakorn.me@up.ac.th; 2 Intelligent and Nonlinear Dynamic Innovations Research Center, Department of Mathematics, Faculty of Applied Science, King Mongkut’s University of Technology North Bangkok, Bangkok 10800, Thailand

**Keywords:** HAR, LSTM, deep learning, time-series data, smartphone sensor, feature extraction

## Abstract

Human Activity Recognition (HAR) employing inertial motion data has gained considerable momentum in recent years, both in research and industrial applications. From the abstract perspective, this has been driven by an acceleration in the building of intelligent and smart environments and systems that cover all aspects of human life including healthcare, sports, manufacturing, commerce, etc. Such environments and systems necessitate and subsume activity recognition, aimed at recognizing the actions, characteristics, and goals of one or more individuals from a temporal series of observations streamed from one or more sensors. Due to the reliance of conventional Machine Learning (ML) techniques on handcrafted features in the extraction process, current research suggests that deep-learning approaches are more applicable to automated feature extraction from raw sensor data. In this work, the generic HAR framework for smartphone sensor data is proposed, based on Long Short-Term Memory (LSTM) networks for time-series domains. Four baseline LSTM networks are comparatively studied to analyze the impact of using different kinds of smartphone sensor data. In addition, a hybrid LSTM network called 4-layer CNN-LSTM is proposed to improve recognition performance. The HAR method is evaluated on a public smartphone-based dataset of UCI-HAR through various combinations of sample generation processes (OW and NOW) and validation protocols (10-fold and LOSO cross validation). Moreover, Bayesian optimization techniques are used in this study since they are advantageous for tuning the hyperparameters of each LSTM network. The experimental results indicate that the proposed 4-layer CNN-LSTM network performs well in activity recognition, enhancing the average accuracy by up to 2.24% compared to prior state-of-the-art approaches.

## 1. Introduction

In the present day, with many countries experiencing aging populations, more elders tend to live alone and are often unable to receive care from family members. It is a recognized fact that elders are subject to falls and accidents when carrying out the activities of daily life. To help single elders live safely and happily, through the Internet of Things (IoT), smart home equipment has been developed to identify the daily activities of elders. In fact, activity recognition is a vital objective in a smart home situation [1]. The ML society is intrigued by Human Activity Recognition (HAR) [2] due to its availability in real-world applications such as fall detection for elderly healthcare monitoring, exercise tracking in sport science [3,4], surveillance systems [5,6,7], and preventing office work syndrome [8]. Currently, HAR is becoming a challenging research topic due to the accessibility of sensors in wearable devices (e.g., smartphone, smartwatch, etc.) which are cost-effective and consume less power, including live cascading of time-series data [9].

Recent research, involving both dynamic and static HAR, uses sensor data collected from wearable devices to better understand the relationship between health and behavioral biometric information [10,11]. The HAR methods can be categorized into two categories according to data sources: visual-based and sensor-based [12]. With visual-based HAR, video or image data are recorded and processed using computer vision techniques [13]. The authors [14] proposes a new approach for identifying sport-related events with video data based on fusion and multiple features. This work achieves high recognition rates in the video-based HAR. Whereas sensor-based HAR works on time-series data captured from a wide range of sensors embedded in wearable devices [15,16]. In [17], the authors researches a context-aware HAR system and notices that an accelerometer is mostly adequate to detect simple activities including walking, sitting, and standing. As adding gyroscope data, the system recognition performance will be increased for employing more complex activities such as drinking, eating, and smoking. However, there are some works to explore other sensors. Fu et al. [18] indicate to improve a HAR framework by using a sensor data of air pressure system along with inertial measurement unit (IMU). This HAR model shows at least 1.78% higher recognition performance than others that is not appliable to sensor data. In the last decade, there is a generational shift in HAR study from device-bound strategies to device-free approaches. Cui et al. [19] introduce a WiFi-based HAR framework by using channel state data to recognize common activities. However, WiFi-based HAR is capable of detecting basic behaviors only, such as running and standing. The cause is that CSI cannot have enough knowledge to understand dynamic events [20].

Sensor-based HAR is becoming more commonly used in smart devices since, with the advancement of pervasive computer and sensor automation, smartphones and their privacy are well protected. Therefore, smartphone sensor-based HAR is the focus of this study. As a wearable device, modern smartphones are becoming increasingly popular. Furnished with an assortment of implanted sensors such as accelerometers, gyroscopes, Bluetooth, and ambient sensors, smartphones also allow researchers to study the activities of daily life. Sensor-based HAR on a device can be considered as an ML model, built to constantly track the actions of the user, despite being connected to a person’s body. Traditional methods have made major strides through the implementation of state-of-the-art deep-learning techniques, including decision tree, naïve Bayes, support vector machine [21], and artificial neural networks [22]. Nevertheless, these traditional ML methods may eventually focus on heuristic, handcrafted feature extraction, which is typically constrained by human domain expertise. However, the efficiency of traditional ML methods is constrained in terms of sorting accuracy and other measurements.

Here, Deep Learning (DL) methods are employed to moderate the previously mentioned limitations. Using multiple hidden layers instead of manual extraction through human domain knowledge allows raw sensor data features to be learned spontaneously. The mining of appropriate in-depth, high-level features for dealing with complex issues such as HAR is facilitated by the deep architecture of these approaches. These DL approaches are now being used to construct a resilient smartphone-based HAR [23,24].

The Convolutional Neural Network (CNN) is a potential DL approach which has achieved favorable results in speech recognition, image classification, and text analysis [25]. When applied to time-series classification-related HAR, the CNN has superiority over other conventional ML approaches, due to its local dependency and scale invariance [26]. Studies on one-dimensional CNNs have shown that these DL models are more effective in solving the HAR problem with performance metrics than conventional ML models [27]. Due to the temporal dependency of sensor time-series data, LSTM networks are introduced to tackle the issue. The LSTM network can identify relationships in the temporal knowledge dimension without combining the time steps as in the CNN network [28].

Ullah et al. [29] proposed a Stacked LSTM network, trained along with accelerometer and gyroscope data, inspired by the emerging DL techniques for sensor-based HAR. These researchers found that recognition efficiency could be enhanced using the Stacked LSTM network to repeatedly extract temporal features. Zhang et al. [30] proposed a Stacked HAR model based on an LSTM network. The findings revealed that with no extra difficulty in training, the Stacked LSTM network could enhance recognition accuracy. Better recognition performance was achieved by combining the CNN network with the LSTM network, based on the study by Mutegeki et al. [31] who used the robustness of CNN network feature extraction while taking advantage of the LSTM model for the classification of time series. To provide promising results in recognition performance, Ordóñez and Roggen [32] combined the convolutional layer with LSTM layers. In order to capture diverse data during training, Hammerla et al. [33] compared different deep neural networks in HAR, including CNN and LSTM, and significantly improved the performance and robustness of recognition. However, existing practices have their own weaknesses and involve various sample generation methods and validation protocols, making them unsuitable for comparison.

To better understand LSTM-based networks for solving HAR problems, this research aims to study LSTM-based HAR using smartphone sensor data. Five LSTM networks were comparatively researched to evaluate the impact of using different kinds of smartphone sensor data from a public dataset called UCI-HAR. Moreover, Bayesian optimization is utilized to manipulate LSTM hyperparameters. Therefore, the primary contributions of this research are as follows:

- A 4-layer CNN-LSTM is proposed: a hybrid DL network consisting of CNN layers and an LSTM layer with the ability to automatically learn spatial features and temporal representation.
- The various experimental results demonstrate that the proposed DL network is suitable for HAR through smartphone sensor data.
- The proposed framework can improve the recognition operation and outperform other baseline DL networks.

The remainder of the paper is structured as follows. Section 2 provides details of the preliminary concept and background theory used in this study. Section 3 presents the proposed HAR framework for obtaining smartphone sensor data. Section 4 shows the experimental conditions and results. The derived results are then discussed in Section 5. Finally, Section 6 presents the conclusion.

## 2. Theoretical Background

### 2.1. HAR from Sensor Data

Generally, HAR systems aim to (1) determine (both online and offline) the ongoing actions/activities of a person, a group of persons, or even a crowd, based on sensory observation data; (2) determine personal characteristics such as the identity of people in a given space, gender, age, etc.; (3) knowledge of the context within which the observed activities are taking place [34]. Therefore, general human activities can be determined as a set of actions performed by a person over a certain period according to a given protocol. It is assumed that a person performs some types of activities by applying a predefined activity set *A* [26]:

(1) $$A=\{a_{1},a_{2},a_{3},\ldots ,a_{m}\}$$

where *m* represents the number of activity classes. Then, a data sequence from sensors reading (*s*) gathers the activity data:

(2) $$s=\{d_{1},d_{2},d_{3},\ldots ,d_{n}\}$$

where $d_{t}$ represents the data reading from sensor at time *t* of a number of sensor data *n*, while *n* ≥ *m*.

The HAR work is to construct the recognition function *F* for predicting the activity sequence based on the data reading *s* of a sensor.

(3) $$F\left(s\right)=\left\{a_{1}^{{}^{\prime }},a_{2}^{{}^{\prime }},a_{3}^{{}^{\prime }},\ldots ,a_{n}^{{}^{\prime }}\right\},a_{i}^{{}^{\prime }}\in A,$$

while a sequence of actual activity is mean as

(4) $$F\left(s\right)=\left\{a_{1}^{*},a_{2}^{*},a_{3}^{*},\ldots ,a_{n}^{*}\right\},a_{i}^{*}\in A.$$

**Figure 1:**
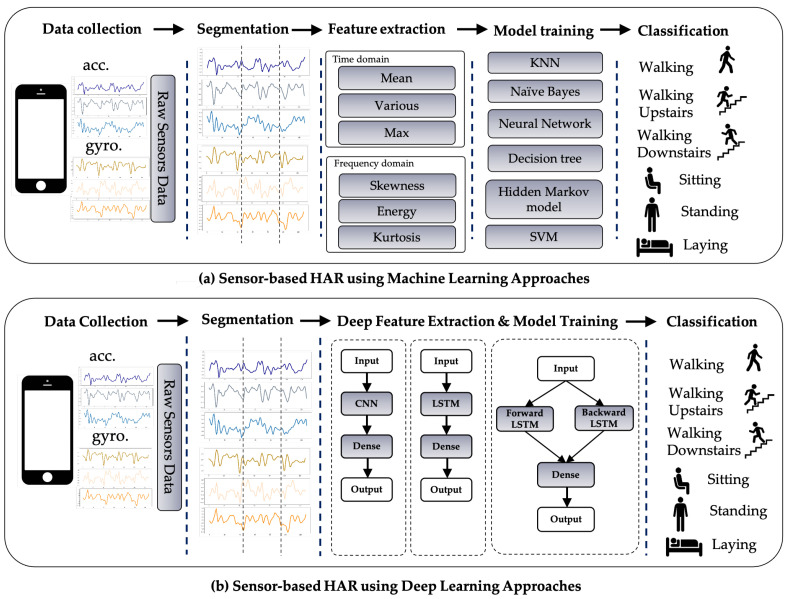
Sensor-based HAR approaches using (**a**) ML technique and (**b**) the DL technique.

Commonly, a HAR system is developed in five fundamental steps: Data collection, Segmentation, Feature extraction, Model training, and Classification, as shown in Figure 1a.

A detailed explanation of each process is designated in the following. The first step in the HAR process is to continuously capture sensor data from a wearable device while the participant is performing predefined activities. The raw sensor data obtained from wearable devices must be commonly pre-processed to remove unwelcome noise. Since the sensor data is represented in time-series format, it must be divided into segments of equal length with a defined window size and a proportion of overlap. Feature extraction is deemed to be the most important step because it defines the operation of the recognition model, and either conventional ML algorithms or DL techniques may be used in this step. Using conventional ML in the time and frequency domain, experts can carefully extract heuristic or handcrafted functions. Numerous time-domain characteristics are available, such as correlation, max, min, mean, standard derivation, etc. A range of frequency-domain characteristics is also available, such as energy, entropy, time between peaks, etc. However, in both domains, handcrafted features have certain drawbacks since they are based on knowledge of the domain and human condition. Such expertise could assist with a specific issue in a unique setting, but cannot be extended to include distinct parameters with the same problem. Moreover, human experience is specifically employed to derive handmade characteristics [35], such as statistical evidence, but refuses to differentiate between events with identical patterns such as standing and sitting behaviors. Some studies have used the methodological approach in ML to construct HAR on smartphones [36,37,38].

DL can help to avoid the drawbacks encountered with role extraction in traditional ML [39]. Figure 1b explains how DL with multiple forms of networks can be applied to HAR. In the DL approach, the feature extraction and model training operations are concurrent. Whereas in the traditional ML approach, the functions can be learned dynamically through the network rather than being individually assembled.

### 2.2. LSTM Networks

Nowadays, LSTM networks [40] are giving an impressive performance across diverse temporal schemes. The LSTM is one of the expanding Recurrent Neural Networks (RNNs). Afterward, their remarkable architecture which actions the dividing gradient problems, LSTM is satisfying at dealing with time-series classification issues.

**Figure 2:**
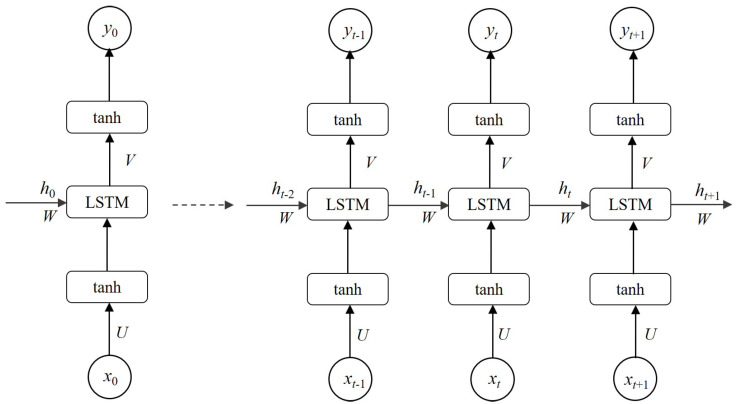
The unfold architecture of one-layer standard LSTM.

The input set is defined as *X* = {$x_{0}$, $x_{1}$, $x_{2}$, …, $x_{t}$, $x_{t+1}$, …}, the output set as *Y* = {$y_{0}$, $y_{1}$, $y_{2}$, …, $y_{t}$, $y_{t+1}$, …}, and the hidden layers as *H* = {$h_{0}$, $h_{1}$, $h_{2}$, …, $h_{t}$, $h_{t+1}$, …}. Then, *U*, *W*, and *V* represent the weight metrics of each layer. *U* represents the values of the weight metrics from the input layer to the hidden layer, *W* represents the values of the weight metrics from the hidden layer to another hidden layer, and *V* represents the values of the weight metrics from the hidden layer to the output layer. The computing mechanism of the LSTM network can hereinafter be summarized. The input data is processed and transformed to the hidden layer using a matrix transformation, accompanied by the hidden layer’s data in the last step. The output data of the hidden layer then passes through an activation function to become the concluding value in the output layer. These detailed processes are illustrated in Figure 2.

Results of hidden layers and output layers can be formally determined as:

(5) $$h_{i}=\left\{\begin{array}{cc} \tanh(Ux_{i}+b_{i}^{h}) & \mathrm{while}\;i\;=\;0\\ \tanh(Ux_{i}+Wh_{i-1}+b_{i}^{h}) & \mathrm{while}\;i\;=\;1,\;2,\;3,\;\ldots \end{array}\right.$$

(6) $$y_{i}=\tanh\left(Vh_{i}+b_{i}^{y}\right)\;\;\;\mathrm{while}\;i\;=\;0,\;1,\;\ldots$$

where *X* = {$x_{0}$, $x_{1}$, $x_{2}$, …, $x_{t}$, $x_{t+1}$, …} is the input set.

**Figure 3:**
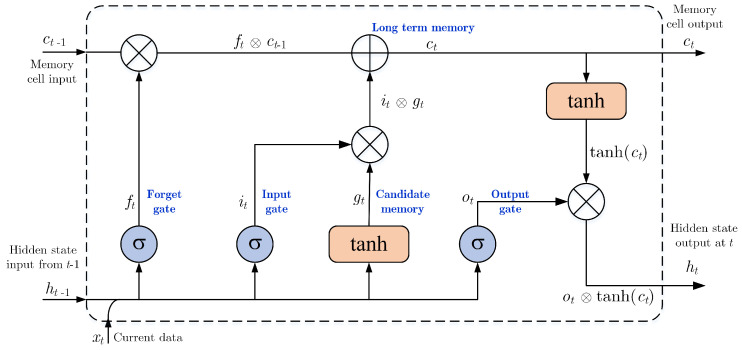
The structure of an LSTM neuron.

In the DL technique, RNNs can predict the current time output based on prior information. However, Bengio et al. [41] inform that RNN networks can recognize the data for only a moment, owing to the dissolving gradient issue. While the deep network backpropagation technique is applied, gradients will be dissolved if permitting gradients to flow deeply are not taken. Hochreiter and Schmidhuber [42] introduced a new neuron into the RNN family, named LSTM, to tackle the problem of long-term dependency. In comparison to the input combination and processing used in RNNs, LSTMs gain the appropriate architecture for recognizing data as an input gate for longer. A forget gate compares the inner memory with new data to overwrite it. This process allows gradients to flow efficiently through time. The input gate, forget gate, output gate, and a memory cell of LSTM (defined as *i*, *f*, *o*, and *C*, respectively) are arranged to manipulate the data which should be disremembered, recognized, and restored as described in Figure 3. The gating technique is chosen to carry the required data. This approach consists of both an activated function (sigmoid function) and an element-wise multiplication function. The output value should be within [0, 1] to enable the multiplication to proceed, and subsequently to allow data to flow (or not). Satisfactory operation can be achieved by assigning the related initialize gates a value of 1 (or close to 1), so training at the beginning is not diminished. Individual criterion in the LSTM neuron at point *t* can be described as follows.

The forget gate $f_{t}$ is accountable for gathering prior data. Then, the recurrent input $h_{t-1}$ and current input $x_{t}$ are multiplied by their weights for being input of a sigmoid function: σ(x) = ${\left(1+e^{-x}\right)}^{-1}$. The output $f_{t}$ is a number within [0, 1] which is multiplied with cell state ($c_{t-1}$). If value output $f_{t}$ is 1, the LSTM will hold this new data, otherwise, if $f_{t}$ = 0 the LSTM will disremember absolutely this data:

(7) $$f_{t}=\sigma \left(U_{f}x_{t}+W_{f}h_{t-1}+b_{f}\right)$$

After that, the input gate $i_{t}$ consists of a sigmoid function. It presents output if $i_{t}$ defines the ongoing value to restore the LSTM cell:

(8) $$i_{t}=\sigma \left(U_{i}x_{t}+W_{i}h_{t-1}+b_{i}\right)$$

The state gate $g_{t}$ builds different values of a vector that are consolidated with a status restore:

(9) $$g_{t}=\tanh\left(U_{g}x_{t}+W_{g}h_{t-1}+b_{g}\right)$$

The output gate $o_{t}$ defines data from the cell state that should appear instantly and associates with previous data:

(10) $$o_{t}=\sigma \left(U_{o}x_{t}+W_{o}h_{t-1}+b_{o}\right)$$

The update state $c_{t}$ consists of disappeared data what is to be forgotten:

(11) $$c_{t}=f_{t}⨂c_{t-1}⨁i_{t}⨂g_{t}$$

The hidden output $h_{t}$ of LSTM composes of short and long terms with:

(12) $$h_{t}=o_{t}⨂\tanh\left(c_{t}\right)$$

The computational mechanism of LSTM cell is detailed in Equations (7)–(12). Firstly, there is a requirement to disremember previous data that relates to the forget gate. The next process is to define new suitable data to hold in memory with an input gate. The old cell state, $c_{t-1}$, to the latest cell state, $c_{t}$ are to be restored as possible. The last step, proper data are determined to be output of the layer above with an output gate.

Recent research studies have proposed a variety of LSTM networks to tackle the problem of time-series classification in HAR. One such network has a simple LSTM configuration called Vanilla LSTM to differentiate it from deeper LSTMs and the suite of more elaborate configurations. This original LSTM architecture was defined by [42], and will give good results on most small sequence prediction problems. The Vanilla LSTM is defined as: the input layer which is fully connected to the hidden layer and output layer of LSTM. The Vanilla LSTM is proposed to overcome the HAR problem [43]. Later, different architecture-based LSTM networks were proposed to solve the HAR problem such as deep LSTM networks called stacked-LSTMs [29], hybrid LSTM networks called CNN-LSTM; combining the CNN with the LSTM [31], mixed LSTM networks called ConvLSTM [44], and bidirectional LSTM networks called Bidir-LSTM [28,45].

## 3. Proposed Methodology

**Figure 4:**
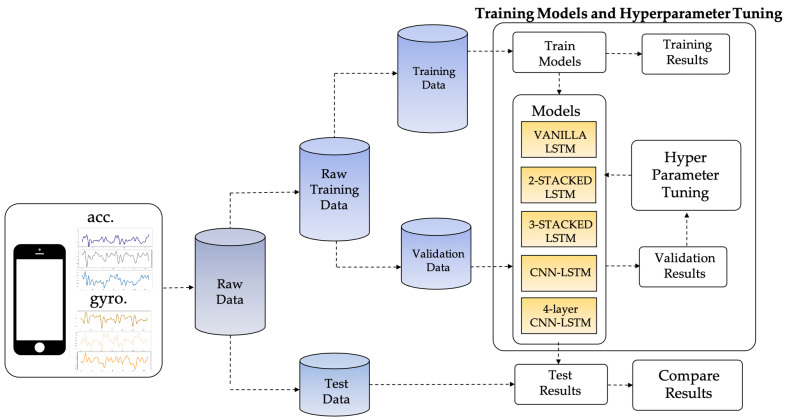
The proposed framework of LSTM-based HAR.

The proposed LSTM-based HAR framework in this study enables the sensor data captured from the smartphone sensor to classify the activity performed by the smartphone user. Figure 4 illustrates the overall methodology used in this study to achieve the research goal. To enhance the recognition efficiency of LSTM-based DL networks, the proposed LSTM-based HAR is presented. Raw sensor data is split into two main subsets during the first stage: raw training data and test data. In the second stage of model training and hyperparameter tuning, the raw training data is further split into 75% for training and 25% for validating the trained model. Five LSTM-based models are tested by the validation data, and the Bayesian optimization approach then tunes the hyperparameters of the trained models. Finally, the hyperparameter-tuned models will be checked against the test results and their recognition performance compared.

### 3.1. UCI-HAR Smartphone Dataset

**Figure 5:**
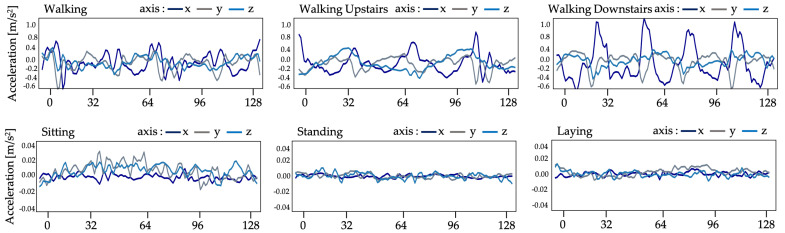
Accelerometer data from UCI-HAR dataset.

**Figure 6:**
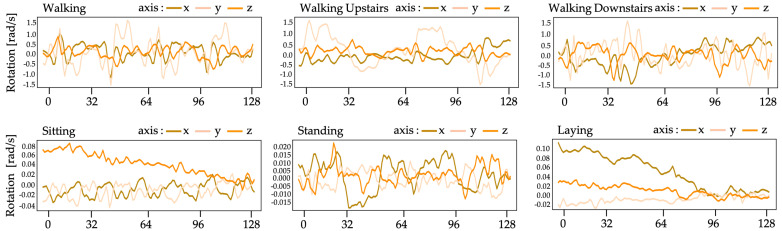
Gyroscope data from UCI-HAR dataset.

The recommended system in this work uses UCI human behavior recognition through a mobile dataset [36] to monitor community activities. Activity data gained from 30 participants of varying ages, races, heights, and weights (aged between 18 and 48 years) was included in the UCI-HAR dataset. While holding a Samsung Galaxy S-II smartphone (Suwon, Korea) at waist level, the subjects carried out everyday tasks. Each person conducted six tasks (i.e., walking, walking upstairs, walking downstairs, sitting, standing, and lying down). The combined tri-axial values of the smartphone accelerometer and gyroscope were used to record sensor data, while the six preset tasks were performed by each of the participants. At a steady rate of 50 Hz, tri-axial values of linear acceleration and angular velocity data were obtained. A detailed description of the UCI-HAR dataset is provided in Table 1. Figure 5 and Figure 6 show the accelerometer and gyroscope data samples, respectively.

**Figure 7:**
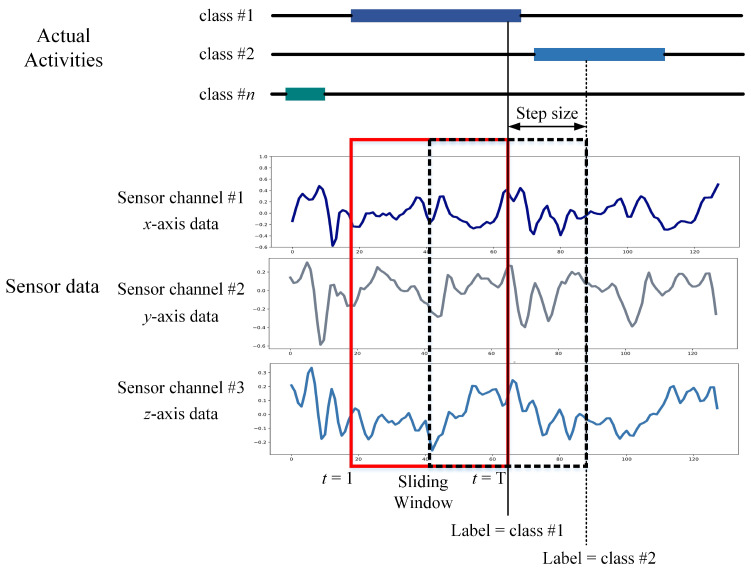
Data segmentation process by a sliding window.

**Figure 8:**
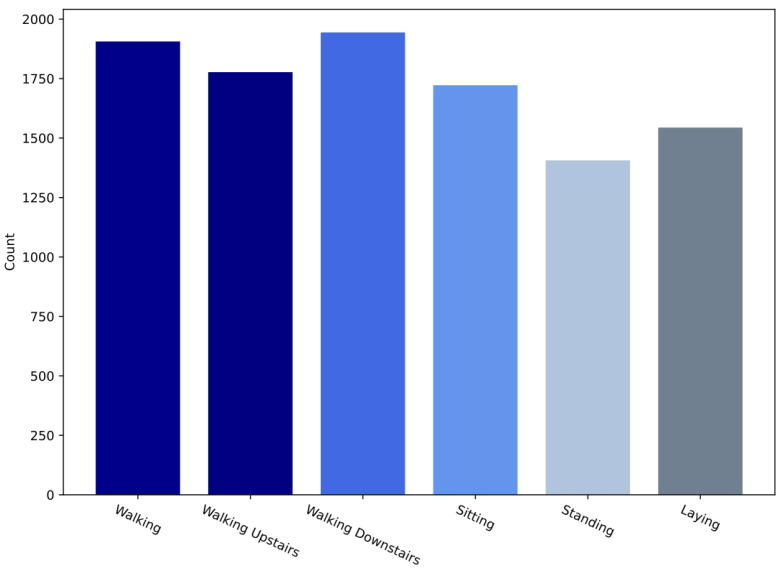
Activity label distribution of UCI-HAR dataset.

An intermediate filter was applied for sound quality pre-processing of the sensor data in the UCI-HAR dataset. A third-order Butterworth low-pass filter with a cutoff frequency of 20 Hz is sufficient for capturing human body motion since 99% of its energy is contained below 15 Hz [46]. The sensor information was then sampled in 2.56 s fixed-width sliding windows with a 50% overlap between them as shown in Figure 7. The four reasons for choosing this window size and overlapping proportion [36] are as follows: (1) The rate of walking of an average person is between 90 and 130 steps/min [47], i.e., a minimum of 1.5 steps/s. (2) For each window study, at least one complete walking period (two steps) is desired. (3) This approach can also help people with a slower cadence, such as the elderly and those with disabilities. A minimum speed equal to 50% of the average human cadence was assumed by the researchers [36]. (4) Signals were also mapped via the Fast Fourier Transform (FFT) in the frequency domain, optimized for two-vector control (2.56 s × 50 Hz = 128 cycles). The available dataset contains 10,299 samples, split into two classes (i.e., two sets of training and testing). The former has 7352 samples (71.39%), while the latter has the remaining 2947 samples (28.61%). The dataset is imbalanced, as shown in Figure 8. Since the use of accuracy only is insufficient for analysis and fair comparison, we additionally apply the F1-score to compare the performance of LSTM-based networks in this work.

**Figure 9:**
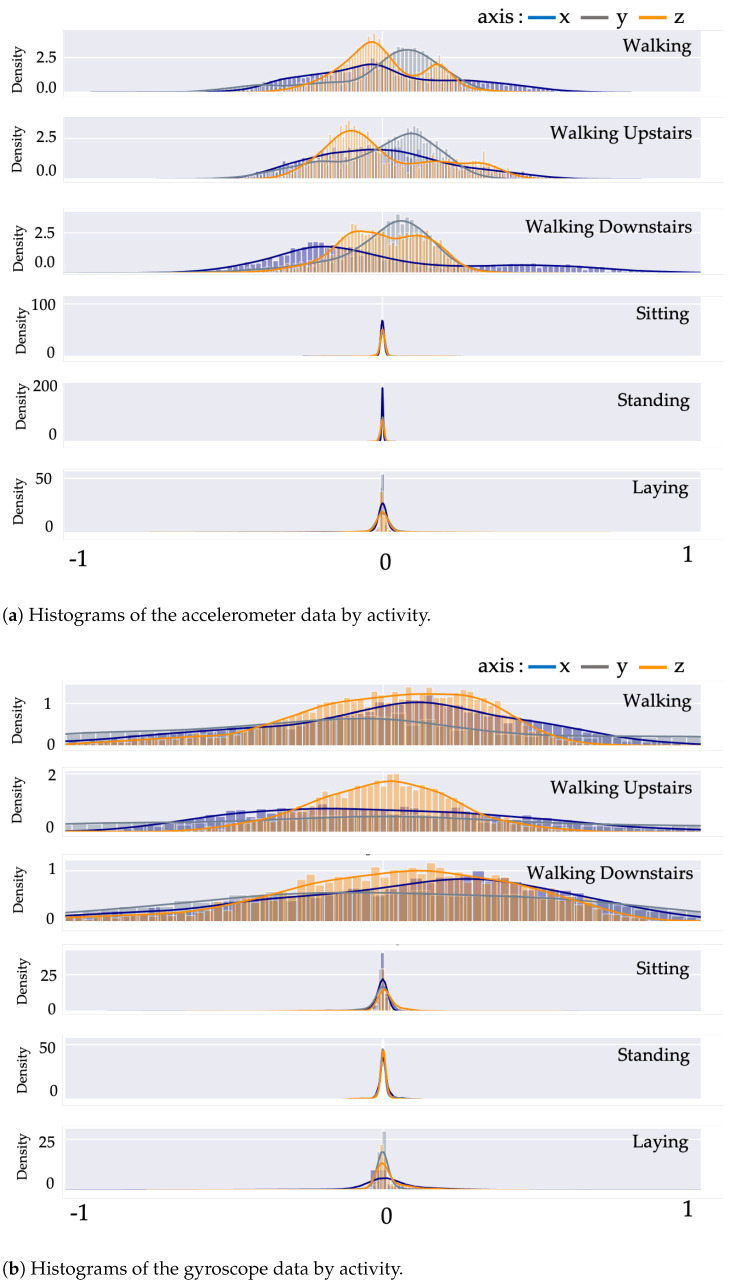
Histograms visualization of data from (**a**) accelerometer (**b**) gyroscope.

To evaluate the DL models, the dataset is first standardized. After employing the normalization approach, the dataset shows zero mean and unit variance. Figure 9 displays the histogram activity data from the tri-axial values of both the accelerometer and gyroscope.

### 3.2. LSTM Architectures

**Figure 10:**
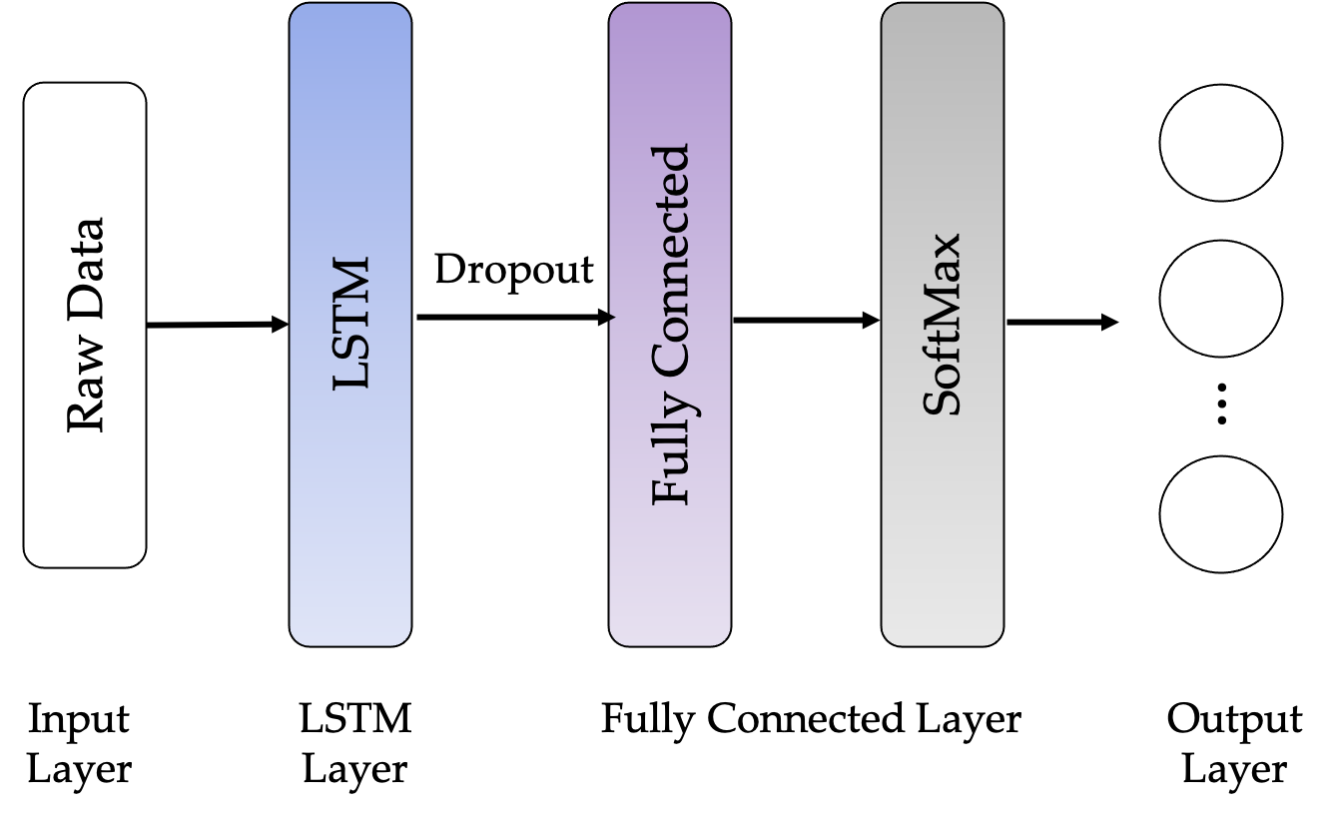
Vanilla LSTM network architecture.

**Figure 11:**
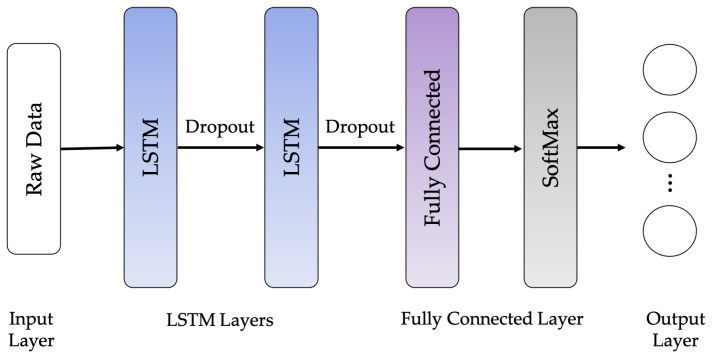
2-Stacked LSTM network architecture.

**Figure 12:**
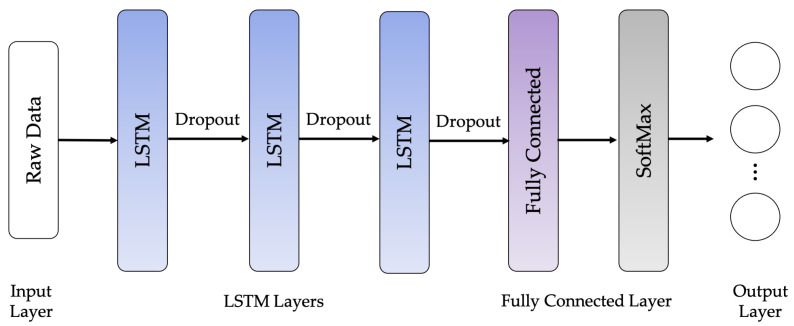
3-Stacked LSTM network architecture.

The following LSTM network architectures are used in this work: Vanilla LSTM network, 2-Stacked LSTM network, and 3-Stacked LSTM network, as illustrated in Figure 10, Figure 11 and Figure 12, respectively. The original LSTM model (or Vanilla LSTM network) comprises an individual hidden layer of LSTM, followed by a common feedforward output layer. The Stacked LSTM networks are upgraded versions of the original model with multiple hidden LSTM layers. Each layer of the Stacked LSTM network contains multiple memory cells. A Stacked LSTM structure can be technologically defined as an LSTM model, consisting of multiple LSTM layers to take advantage of the temporal feature extraction obtained from each LSTM layer.

**Figure 13:**
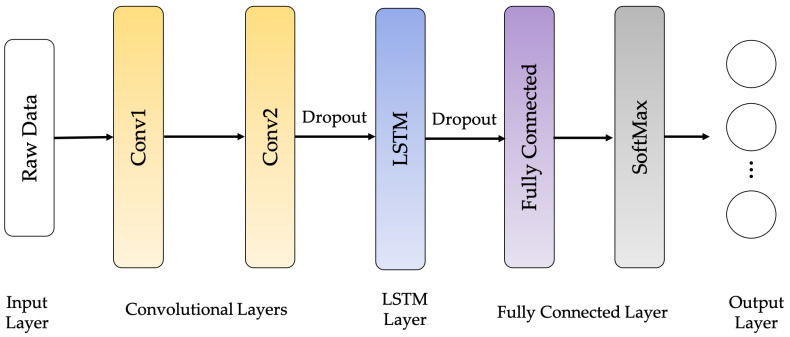
CNN-LSTM network architecture.

**Figure 14:**
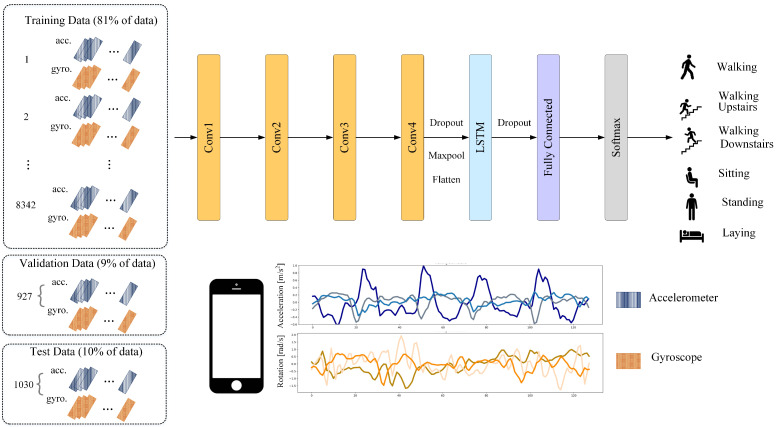
The proposed architecture of 4-layer CNN-LSTM network.

The CNN-LSTM architecture employs CNN layers in the feature extraction process of input data incorporated with LSTMs to support sequence forecasting, as shown in Figure 13. The CNN-LSTMs are built to solve forecasting problems in visual time series and applications to achieve textual descriptions from image sequences. This architecture is appropriate for issues involving a temporal input structure or requiring output generation with a temporal structure. In this work, an LSTM network called 4-layer CNN-LSTM is proposed to improve recognition performance. The architecture of the proposed 4-layer CNN-LSTM is illustrated in Figure 14.

The network architecture of the proposed 4-layer CNN-LSTM network is shown in Figure 14. The tri-axial accelerometer and tri-axial gyroscope data segments were used as network inputs. To extract feature maps from the input layer, four one-dimensional convolutional layers were used for the activation feature in ReLU. Then, to summarize the feature maps provided by the convolution layers and reduce the computational costs, a max-pooling layer is also added to the proposed network. Their dimensions also need to be reduced after reducing the size of the function maps to allow the LSTM network to operate. For this reason, the flattened layer converts each function map’s matrix representation into a vector. In addition, several dropouts are inserted on top of the pooling layer to decrease the risk of overfitting.

The output of the pooling layer is processed by an LSTM layer after the dropout function is applied. This models the temporal dynamics to trigger the feature maps. A fully connected layer, followed by a SoftMax layer to return identification, is the final layer. Hyperparameters such as filter number, kernel size, pool size, and dropout ratio were determined by Bayesian optimization, as shown in Figure 3.

### 3.3. Tuning Hyperparameter by Bayesian Optimization

Hyperparameters are essential for DL approaches since they directly manipulate the actions of training algorithms and have a crucial effect on the performance of DL models. Bayesian optimization is a practical approach for solving the function problems prevalent in computing for finding the extrema. This approach is suitably employed for solving a related-function problem where the expression has no closed form. Bayesian optimization can also be applied to related-function problems such as extravagant computing, hard derivative evaluation, or a non-convex function. In this work, the optimization goal is to discover the maximum value for an unknown function *f* at the sampling point:

(13) $$x^{+}=\arg\max_{x\in A}f\left(x\right)$$

where *A* represents the search space of *x*. Given evidence data *E* are derived from Bayes’ theorem of Bayesian optimization. Then, the posterior probability *P(M|E)* of a model *M* is comparable to the possibility *P(E|M)* of over-serving *E* given model *M* multiplied by the prior probability of *P(M)*:

(14) $$P\left(M|E\right)=\frac{P(E|M)P(M)}{P(E)}$$

The Bayesian optimization technique has recently become popular as a suitable approach for tuning deep network hyperparameters by reason of their capability in administering multi-parametric issues with valuable objective functions when the first-order derivative is not applicable.

### 3.4. Sampling Generation Process and Validation Protocol

The first process in sensor-based HAR is to create raw sensor data for the samples. This procedure involves separating it into small windows of the same size, called temporal windows. The raw sensor data is then interpreted as time-series data. Then, as the data samples are separated into training data, the temporal windows from the signals are used to learn a model and test the data to validate the learned model.

There are many strategies for using temporal windows to obtain data segments. The overlapping temporal window (OW), whereby a fixed-size window is applied to the input data sequence to provide data for training and test samples using certain validation protocol, is the most generally used window in sensor-based HAR studies (e.g., 10-fold cross validation). However, this technique is highly biased since there is a 50% overlap between subsequent sliding windows. Another method called the non-overlapping temporal window (NOW) can prevent this bias. As opposed to the OW technique, the NOW has the disadvantage of only a limited number of samples since the temporal windows no longer overlap.

Evaluating the prediction metrics of the trained models is a critical stage of the process. Cross Validation (CV) [48] is used as the standard technique, whereby the data is separated into training and test data. Various approaches, such as leave-one-out, leave-p-out, and k-fold cross validation, can be used to separate the data for training and testing [49,50]. The objective of this process is to evaluate the ability of the learning algorithm to generalize new data [50].

In sensor-based HAR, the purpose is to generalize a model for a different subject. The cross-validation protocol should also be subject-specific, meaning that the training and testing data contain records of various subjects. Cross validation of this protocol is called Leave-One-Subject-Out (LOSO). In order to create a model, the LOSO employs samples of all subjects but leaves one out to shape the training data. Then, using the samples of the excluded subject, the trained model is examined [51].

In this work, four combinations of sample generation processes and validation protocols evaluate the output recognition of LSTM networks, as shown in Table 2.

### 3.5. Performance Evaluation Metrics

The LSTM network classifiers employed in HAR can be measured using a few performance assessment indices. Five assessment metrics: accuracy, precision, recall, F1-score, and AUC are chosen for performance evaluation.

The accuracy metric represents the corrected ratio of forecasting samples to total samples. It is suitable for assessing the classification performance in the case of balanced data. On the other hand, the F1-score represents the weighted average of both precision and recall. Therefore, this metric can be properly applied in the event the data is imbalanced. Six metrics can be mathematically defined as the following expressions.

1. The accuracy is the evaluation ratio metric to all true assessment results of summarize the total grouping achievement for all types:
  (15) $$\mathrm{Accuracy}=\frac{True_{P}+True_{N}}{True_{P}+False_{P}+True_{N}+False_{N}}$$
2. The precision represents the ratio of positive samples classified correctly to total positive samples:
  (16) $$\mathrm{Precision}=\frac{True_{P}}{True_{P}+False_{P}}$$
3. The recall represents the ratio of positive samples classified correctly to total positive samples:
  (17) $$\mathrm{Recall}=\frac{True_{P}}{True_{P}+False_{N}}$$
4. The F1-score represents the union of the recall value and the precision value in a separated value:
  (18) $$F1-\mathrm{score}=2\times \frac{\mathrm{Recall}\times \mathrm{Precision}}{\mathrm{Recall}+\mathrm{Precision}}$$
5. Receiver Operating Characteristics (ROC) Curve, known as precision-recall rate, presents an approach for determining the true positive rate (TPR) against the false positive rate (FPR):
  (19) $$\mathrm{TPR}=\frac{True_{P}}{True_{P}+False_{P}}$$
  The false positive rate refers to the proportion of positive data points which are inappropriately claimed to be negative in comparison to all negative data points.
  (20) $$\mathrm{FPR}=\frac{False_{P}}{True_{N}+False_{P}}$$
  where $True_{P}$ means the number of true positives, $True_{N}$ means the number of true negatives, $False_{P}$ means the number of false positives, and $False_{N}$ means the number of false negatives.
  Not only Area Under the Curve (AUC) indicates the overall efficiency of classifiers, but it also describes the likelihood that positive cases selected will be ranked higher than negative cases [34].

## 4. Experiments and Results

In the experimental section, the process is described and the results used to evaluate the LSTM networks for HAR.

### 4.1. Experiments

**Figure 15:**
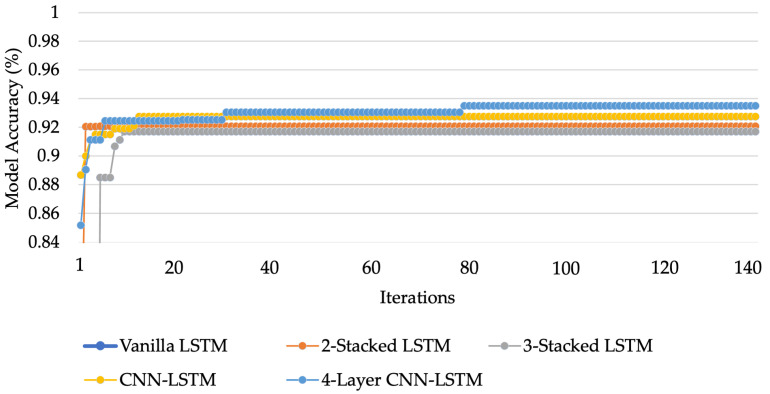
The accuracy of each model after optimization process.

**Figure 16:**
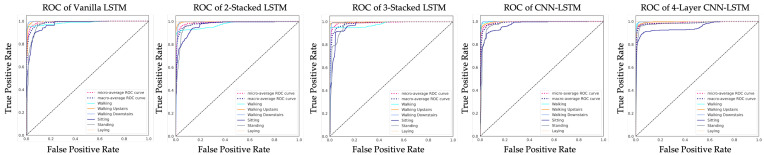
Receiver operating characteristic curves of five LSTM models.

To compare the performance of each LSTM network and address the HAR issue, the experiments are varied. For the first variation, a basic LSTM network called Vanilla LSTM network is used, composed of only one LSTM hidden layer with dropout and one dense layer. For the second variation, one more LSTM hidden layer is added. This kind of configuration is called 2-Stacked LSTM. In the third variation, as well as adding an LSTM hidden layer, a 3-Stacked LSTM is included. The fourth variation is the CNN-LSTM; an LSTM network combined with a convolution layer to create an LSTM layer. In process of the hyperparameter optimization, the fifth LSTM-based networks were tuned by Bayesian optimization algorithm. The accuracy of each model after optimization is shown in Figure 15. After hyperparameter tuning, the 4-layer CNN-LSTM achieves an accuracy of 93.519% that outperforms other models after 140 iterations. The hyperparameters for the Vanilla LSTM network, 2-Stacked LSTM network, 3-Stacked LSTM network, CNN-LSTM network, and the proposed 4-layer CNN-LSTM network are summarized in Table 3, Table 4, Table 5, Table 6 and Table 7, respectively. The ROC of five LSTM models determining the ability of each classifier model is shown in Figure 16.

### 4.2. Results

The results derived from the conducted experiments are presented in this section. The experimental hardware and software configurations are as follows. The LSTM-based HAR models are implemented using Python’s Scikit-Learn, TensorFlow [52] Core v2.0.0-rc0, and Keras v2.4.0 with each test consisting of different LSTM models. All experiments were executed using Tesla K80 GPU (NVIDIA, USA) on the Google Colab platform.

Furthermore, the hyperparameters of LSTM networks were optimized using the Bayesian hyperparameter optimization on the SigOpt platform [53]. This is a scalable and regulated platform accompanied by an API to facilitate the generation of well-performing models. It also allows the process to proceed in parallel for faster evaluation.

The LSTM networks trained on the UCI-HAR dataset were evaluated using two different sample generation processes and two validation protocols, 10-fold and LOSO cross validation. Several experiments were conducted to evaluate the recognition performance of the LSTM networks with a set of evaluation indices: accuracy, precision, recall, F1-score, and AUC. Table 8 shows the accuracy derived from the various LSTM networks trained on the UCI-HAR dataset.

The accuracy, precision, recall, F1-score, and AUC of different LSTM networks are shown in Table 8 and Table 9 using two different sample generation procedures (OW and NOW, respectively) and evaluated by the 10-fold protocol of cross validation. As can be observed from both tables: (1) The accuracy in all five recognition models was greater than 95%, with F1 scores greater than 99%. This means that for the six regular operations, the proposed HAR system could accurately identify behavior. (2) In all five examples, the precision, recall, and F1-scores outperformed the accuracy level. The accuracy metrics not only take $True_{P}$ and $False_{P}$ into calculation, but also $True_{N}$ and $False_{N}$, by comparing Equations (15)–(18). Lower precision means that the $True_{N}$ is substantially less than $False_{N}$ and $False_{P}$. In other words, the most positive samples can be found in all LSTM networks. (3) All the evaluation metrics of the five OW process data-trained LSTM networks were greater than those of the NOW process. This shows that the amount of data affected the recognition efficiency of the five LSTM networks, but the difference was not statistically significant. (4) With a high accuracy of 99.39% from the OW process and 98.76% from the NOW process, the proposed 4-layer CNN-LSTM network outperformed other LSTM-based networks.

The performance metrics (accuracy, precision, recall, F1-score, and AUC) of various LSTM-based networks using two separate sample generation processes (OW and NOW) are shown in Table 10 and Table 11, evaluated by the leave-one-subject-out cross-validation protocol. As can be observed from the experimental results in Table 10: (1) The accuracy of all five LSTM-based models was greater than 92% and F1 score greater than 75%. The highest average precision was 96.70% and the highest F1-score 81.05%, indicating that the proposed 4-layer CNN-LSTM was achieved. (2) In all five situations, the precision, recall, and F1-score outperformed the accuracy level. The precision metrics not only take $True_{P}$ and $False_{P}$ into calculation, but also $True_{N}$ and $False_{N}$, by comparing the metric Equations (15)–(18). Higher accuracy indicates that $True_{N}$ was substantially greater than $False_{N}$ and $False_{P}$. This means that most negative samples could be recognized by all LSTM networks. It can be observed from the experimental results in Table 11 that the proposed 4-layer CNN-LSTM outperformed other LSTM-based networks with the highest accuracy of 93.94%. The confusion matrix of the proposed 4-layer CNN-LSTM networks is illustrated in Table 12.

### 4.3. Comparative Results

Table 8, Table 9, Table 10 and Table 11 show the comparative results between the experimental LSTM hybrid networks and the LSTM baseline network. Since the UCI-HAR dataset is imbalanced, the accuracy is inadequate for valid comparison to be evaluated, so the F1-score is also used to compare the recognition performance of these LSTM networks.

Considering to experimental results on average of 10-fold cross validation by OW sample generation, the derived results demonstrates that hybrid LSTMs has better recognition performance than conventional LSTMs with higher accuracy and F1-score upto 2.13% and 0.11%, respectively. Additional with NOW sample generation, They are also better than conventional LSTMs with 2.77% and 0.24% of accuracy and F1-score, respectively. At the same time with hybrid LSTMs, the proposed 4-layer CNN-LSTM has more performance of activity recognition than CNN-LSTM with 0.90% and 0.11% of accuracy and F1-score in OW sample generation, and 1.32% and 0.44% of accuracy and F1-score for NOW sample generation, respectively.

In LOSO cross validation with results on average, hybrid LSTMs have more recognition performance than conventional LSTMs with 2.45% of accuracy for OW sample generation. Moreover with NOW sample generation, hybrid LSTMs still have more recognition performance than conventional LSTMs with 1.98% and 0.745% of accuracy and F1-score, respectively. Considering to hybrid LSTMs, the proposed 4-layer CNN-LSTM has more performance of activity recognition than CNN-LSTM with 2.58% and 3.28% of accuracy and F1-score in OW sample generation, respectively.

**Figure 17:**
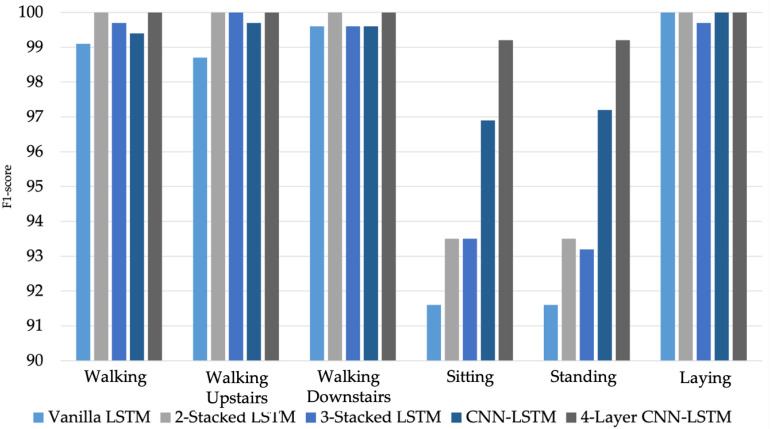
Bar chart showing F1-score of the different LSTM networks on the UCI-HAR dataset.

**Figure 18:**
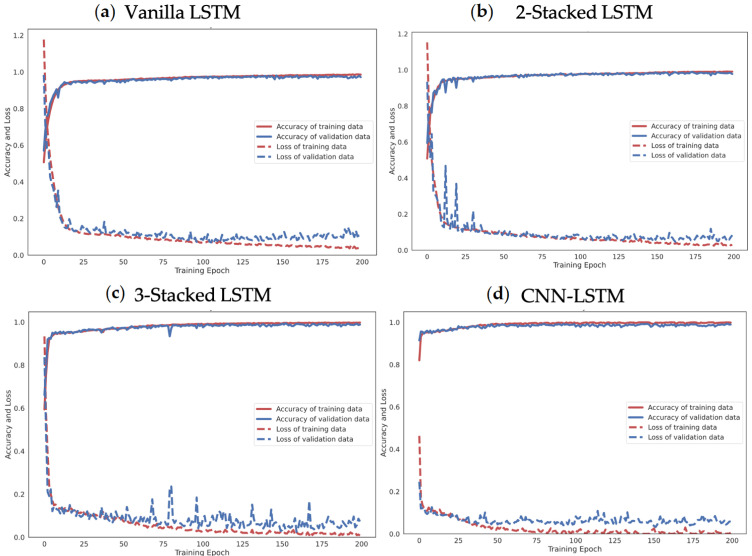
Accuracy and loss examples of training process of Vanilla LSTM, 2-stacked LSTM, 3-stacked LSTM, and CNN-LSTM.

**Figure 19:**
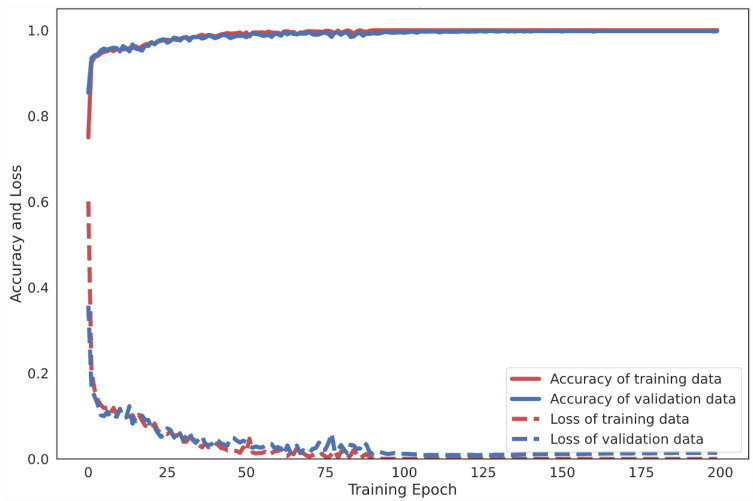
Accuracy and loss examples of training process of the proposed 4-layer CNN-LSTM.

Moreover, the effectiveness of the proposed 4-layer CNN-LSTM with the F1-score of each activity class was compared. The F1-score results of the different LSTM-based DL models trained from smartphone sensor data are shown in Figure 17. The accuracy and loss details of the training process for four models (Vanilla LSTM network, 2-Stacked LSTM network, 3-Stacked LSTM network, and CNN-LSTM network) and the proposed 4-layer CNN-LSTM are illustrated in Figure 18 and Figure 19, respectively.

### 4.4. Comparative Analysis

An accuracy comparison between the proposed model and other LSTM networks is shown in Table 13. The proposed 4-layer CNN-LSTM network outperformed the other previous works. This is because the spatial feature extraction performed by the 4-layer CNN improved the overall accuracy by up to 2.24% compared to the most recent work [54].

## 5. Discussion of Results

The results derived from the experiments presented in Section 4 are the main contributions of this research, and the following discussion is provided to ensure the consequences are clear.

LSTM-based DL networks determined in this study could be employed to precisely categorize activities, specifically typical human daily living activities. The categorized process used tri-axial data on both accelerometers and gyroscopes embedded in smartphones. The categorized accuracy and other advanced metrics were considered to evaluate the sensor-based HAR of five LSTM-based DL architectures. A publicly available dataset of previous studies [25,31,36,45,54] was applied to compare the generality of DL algorithms with the 10-fold cross-validation technique.

The UCI-HAR raw smartphone sensor data were evaluated with the Vanilla LSTM network, 2-Stacked LSTM network, 3-Stacked LSTM network, CNN-LSTM network, and the proposed 4-layer convolutional LSTM network (4-layer CNN-LSTM network). First, activity classification based on sensors with raw tri-axial data from both the accelerometer and gyroscope has been demonstrated. Figure 18a illustrates the process of learning raw sensor data with the Vanilla LSTM network. The loss rate decreased gradually and the accuracy rate increased slowly without any appearance of dilemma. This indicates that the network learns appropriately without overfitting problems. The final result shows an average accuracy of 96.54% with the testing set. Figure 18b shows the training result of the 2-Stacked LSTM network. With the training set, both loss and accuracy were thoroughly trained and provide a decent performance. However, during the testing set process, some bouncing occurred. Specifically, when the epoch was 23, bouncing in both loss and accuracy could obviously be observed. Fortunately, the final average accuracy result is still better than that for the Vanilla LSTM network. During the testing set process, the loss rate was 0.1394 and the average accuracy 97.32%. The training result of the 3-Stacked LSTM network in Figure 18c shows a satisfactory learning process, but the testing set was significantly unstable. There are many bouncing spots in the middle and final epochs. While the epoch was repeated, the loss rate appeared to fluctuate. The process appears in worse shape when considering it as a graph. Nonetheless, the results in the actual testing set did not change for the worse with a loss rate of 0.189 and an accuracy rate of 96.59%. In Figure 18d, the training process of the CNN-LSTM network is shown to generate unstable parts in the validation set. On the other hand, the accuracy is generally higher than when training with the baseline LSTM model, including the 2-Stacked LSTM network and 3-Stacked LSTM network, which achieved 98.49% in the testing set. Figure 19 shows the learning process of the proposed 4-layer CNN-LSTM. In the testing set, with the epoch at 50, the loss rate decreased significantly and the accuracy increased significantly. Both the loss rate and accuracy quickly stabilized. Finally, the accuracy was 99.39% in the testing set.

The derived classification results in this study reveal that hybrid DL architectures can improve the prediction performance of the baseline LSTM. When associating two DL architectures (CNN and LSTM), the hybrid architecture could be the dominant reason for the increased scores, since the CNN-LSTM network produced higher average accuracy and F1-score than the baseline LSTM networks. Therefore, it can be inferred that the hybrid model delivers the advantages of both CNN and LSTM in terms of extracting regional features within short time steps and a temporal structure across a sequence.

A limitation of this study is that the algorithms for deep learning are trained and tested using laboratory data. Previous studies have shown that the performance of learning algorithms could not accurately reflect performance in everyday life under laboratory conditions [55]. Another constraint is that when looking at real-world situations, this study does not discuss the issue of transitional behaviors (Sit-to-Standing, Sit-to-Lay, etc.), which is a challenge goal. However, the proposed HAR architecture can be applicable to many realistic applications in smart homes with high performance deep learning networks including optimal human movement in sports, healthcare monitoring and safety surveillance for elderly people, and baby and child care.

## 6. Conclusions and Future Works

In this study, the LSTM-based framework explores the LSTM network, providing high performance in addressing the HAR problem. Four LSTM networks were selected to study their recognition performance using different smartphone sensors, i.e., tri-axial accelerometer and tri-axial gyroscope. These LSTM networks were evaluated using a publicly available dataset called UCI-HAR by considering predictive accuracy and other performance metrics such as precision, recall, F1-score, and AUC. The experimental results show that the 4-layer CNN-LSTM network proposed in this study outperforms the other baseline LSTM networks with a high accuracy rate of 99.39%. Moreover, the proposed LSTM network was compared to previous works. The 4-layer CNN-LSTM network could improve the accuracy by up to 2.24%. The advantage of this model is that the CNN layers perform direct mapping in the spatial representation of raw sensor data for feature extraction. The LSTM layers take full advantage of the temporal dependency to significantly improve the extraction features of HAR.

Future work would involve the further development of LSTM models using various hyperparameters, including regularization, learning rate, batch size, and others. Furthermore, the proposed model could be applied to more complicated activities to tackle other DL challenges and HAR by evaluating it on other public activity datasets, such as OPPORTUNITY and PAMAP2. 

## Figures and Tables

**Table 1:** Description of UCI-HAR dataset.

Activity	Abbreviates	Description	No. of Samples
Walking	Wa	Participant walks horizontal forward in a direct position	1722
Walking (Upstairs)	Wu	Participant walks upstairs	1544
Walking (Downstairs)	Wd	Participant walks downstairs	1406
Sitting	Si	Participant sits on a chair	1777
Standing	St	Participant stands inactive	1906
Laying	La	Participant sleeps or lies down	1944

**Table 2:** Combination between sample generation processes and validation process used in this study.

Sample Generation Process	Validation Protocol	
	10-Fold CV	Leave-One-Subject-Out CV
Overlapping-Windows	OWCV	OWLS
Non Overlapping-Windows	NOCV	NOLS

**Table 3:** The summarized hyperparameters of Vanilla LSTM network found by SigOpt.

Phase	Hyperparameter	Value
Structure	LSTM-neuron	94
	Dropout	0.28385
	Dense	784
Training	Loss Function	Cross-entropy
	Optimizer	RMSprop
	Batch Size	64
	Learning Rate	1 × 10^−3.5637^
	Number of Epoches	100

**Table 4:** The summarized hyperparameters of 2-Stacked LSTM network found by SigOpt.

Phase	Hyperparameter	Value
Structure	LSTM-neuron${}_{1}$	63
	Dropout${}_{1}$	0.46892
	LSTM-neuron${}_{2}$	39
	Dropout${}_{2}$	0.06469
	Dense	181
Training	Loss Function	Cross-entropy
	Optimizer	RMSprop
	Batch Size	64
	Learning Rate	1 × 10^−3.32288^
	Number of Epoches	191

**Table 5:** The summarized hyperparameters of 3-Stacked LSTM Network found by SigOpt.

Phase	Hyperparameter	Value
Structure	LSTM-neuron${}_{1}$	74
	Dropout${}_{1}$	0.08753
	LSTM-neuron${}_{2}$	43
	Dropout${}_{2}$	0.32057
	LSTM-neuron${}_{3}$	36
	Dropout${}_{3}$	0.30374
	Dense	338
Training	Loss Function	Cross-entropy
	Optimizer	RMSprop
	Batch Size	64
	Learning Rate	1×10 ${}^{-2.84401}$
	Number of Epoches	50

**Table 6:** The summarized hyperparameters of CNN-LSTM network found by SigOpt.

Phase	Hyperparameter		Value
Structure	Convolution${}_{1}$	Kernel-Size	3
		Stride	1
		Filters	39
	Convolution${}_{2}$	Kernel-Size	3
		Stride	1
		Filters	62
	Dropout${}_{1}$		0.02205
	Maxpooling		2
	Dense		83
	Dropout${}_{2}$		0.27907
	Dense		10
Training	Loss Function		Cross-entropy
	Optimizer		Adam
	Batch Size		64
	Learning Rate		1×10 ${}^{-2.67193}$
	Number of Epoches		100

**Table 7:** The summarized hyperparameters for 4-layer CNN-LSTM network found by SigOpt.

Stage	Hyperparameter		Value
Structure	Convolution${}_{1}$	Kernel-Size	3
		Stride	1
		Filters	507
	Convolution${}_{2}$	Kernel-Size	3
		Stride	1
		Filters	111
	Convolution${}_{3}$	Kernel-Size	3
		Stride	1
		Filters	468
	Convolution${}_{4}$	Kernel-Size	3
		Stride	1
		Filters	509
	Dropout${}_{1}$		0.00952
	Maxpooling		2
	LSTM-neuron		127
	Dropout${}_{2}$		0.27907
	Dense		772
Training	Loss Function		Cross-entropy
	Optimizer		Adam
	Batch Size		64
	Number of Epoches		182

**Table 8:** Performance metrics of five LSTM networks used in the experiment by OW sample generation and 10-fold cross validation protocol.

Network	Evaluation Metrics (±std.)				
	Accuracy	Precision	Recall	F1-Score	AUC
Vanilla LSTM network	96.54% (±0.408%)	99.54% (±0.700%)	99.66% (±0.414%)	99.60% (±0.474%)	99.79% (±0.136%)
2-stacked LSTM network	97.32% (±0.608%)	99.93% (±0.182%)	99.43% (±0.603%)	99.68% (±0.289%)	99.63% (±0.135%)
3-stacked LSTM network	96.59% (±0.475%)	99.66% (±0.433%)	99.77% (±0.408%)	99.71% (±0.233%)	99.73% (±0.246%)
CNN-LSTM network	98.49% (±0.751%)	99.90% (±0.205%)	99.55% (±0.609%)	99.72% (±0.313%)	99.78% (±0.088%)
4-layer CNN-LSTM network	99.39% (±0.248%)	99.93% (±0.205%)	99.74% (±0.744%)	99.83% (±0.378%)	99.82% (±0.090%)

**Table 9:** Performance metrics of five LSTM networks used in the experiment by NOW sample generation and 10-fold cross validation protocol.

Network	Evaluation Metrics (±std.)				
	Accuracy	Precision	Recall	F1-Score	AUC
Vanilla LSTM network	94.93% (±0.908%)	99.29% (±1.120%)	99.88% (±0.186%)	99.58% (±0.537%)	99.73% (±0.138%)
2-stacked LSTM network	96.23% (±1.060%)	99.44% (±0.946%)	99.26% (±0.923%)	99.35% (±0.807%)	99.58% (±0.209%)
3-stacked LSTM network	94.85% (±0.820%)	99.59% (±0.713%)	98.95% (±2.455%)	99.25% (±1.377%)	99.73% (±0.115%)
CNN-LSTM network	97.44% (±0.607%)	99.33% (±1.221%)	99.51% (±0.911%)	99.41% (±0.660%)	99.63% (±0.231%)
4-layer CNN-LSTM network	98.76% (±0.500%)	99.96% (±0.119%)	99.73% (±0.800%)	99.85% (±0.403%)	99.65% (±0.244%)

**Table 10:** Performance metrics of five LSTM networks used in the experiment by OW sample generation and LOSO cross validation protocol.

Network	Evaluation Metrics (±std.)				
	Accuracy	Precision	Recall	F1-Score	AUC
Vanilla LSTM network	92.62% (±8.255%)	80.29% (±4.287%)	78.88% (±7.741%)	79.48% (±5.950%)	98.26% (±3.684%)
2-stacked LSTM network	92.52% (±8.773%)	80.56% (±4.363%)	79.74% (±6.620%)	80.08% (±5.259%)	97.65% (±4.318%)
3-stacked LSTM network	93.75% (±6.833%)	80.06% (±5.002%)	79.92% (±6.224%)	79.94% (±5.423%)	98.18% (±3.314%)
CNN-LSTM network	94.12% (±7.625%)	80.16% (±4.990%)	78.10% (±14.824%)	77.77% (±14.333%)	97.82% (±4.236%)
4-layer CNN-LSTM network	96.70% (±3.516%)	80.54% (±5.614%)	81.63% (±5.480%)	81.05% (±5.332%)	98.60% (±1.927%)

**Table 11:** Performance metrics of five LSTM networks used in the experiment by NOW sample generation and LOSO cross validation protocol.

Network	Evaluation Metrics (±std.)				
	Accuracy	Precision	Recall	F1-score	AUC
Vanilla LSTM network	91.61% (±8.868%)	96.65% (±7.717%)	91.37% (±19.815%)	92.37% (±17.103%)	98.17% (±3.413%)
2-stacked LSTM network	92.03% (±8.232%)	95.70% (±8.233%)	94.66% (±11.054%)	94.87% (±8.717%)	97.60% (±4.670%)
3-stacked LSTM network	92.52% (±7.972%)	97.38% (±7.493%)	95.58% (±9.761%)	96.33% (±8.207%)	98.19% (±3.598%)
CNN-LSTM network	94.13% (±7.954%)	97.67% (±7.431%)	95.86% (±9.264%)	96.64% (±7.992%)	98.02% (±4.324%)
4-layer CNN-LSTM network	93.94% (±8.190%)	96.55% (±7.817%)	96.76% (±7.823%)	93.89% (±16.174%)	97.62% (±3.855%)

**Table 12:** Confusion Matrix for the proposed 4-layer CNN-LSTM.

	Walking	Walking${}_{\mathrm{Upstairs}}$	Walking${}_{\mathrm{Downstairs}}$	Sitting	Standing	Laying	Recall
Walking	172	0	0	0	0	0	1.00
Walking${}_{Upstairs}$	0	154	0	0	0	0	1.00
Walking${}_{Downstairs}$	0	0	141	0	0	0	1.00
Sitting	0	0	0	178	0	0	1.00
Standing	0	0	0	3	188	0	0.984
Laying	0	0	0	0	0	194	1.00
Precision	1.000	1.000	1.000	0.983	1.000	1.000	

**Table 13:** Performance comparison results.

Ref.	Architecture	Accuracy	Year
Lee [25]	1D-CNN	92.71%	2017
Hernandez [45]	Bidir-LSTM	92.67%	2019
Mutegeki [31]	CNN-LSTM	92.13%	2020
Ni [54]	SDAE	97.15%	2020
The proposed approach	4-layer CNN-LSTM	99.39%	-

## Data Availability

Not applicable.
